# Overproduction of *Xanthophyll* Pigment in *Flavobacterium* sp. JSWR-1 under Optimized Culture Conditions

**DOI:** 10.4014/jmb.2310.10034

**Published:** 2023-11-24

**Authors:** Jegadeesh Raman, Young-Joon Ko, Jeong-Seon Kim, Da-Hye Kim, Soo-Jin Kim

**Affiliations:** Agricultural Microbiology Division, National Institute of Agricultural Sciences, Rural Development Administration, Wanju-gun, Jeollabuk-do 55365, Republic of Korea

**Keywords:** Carotenoid, zeaxanthin, *Flavobacterium*, HPLC, pigments, antioxidant

## Abstract

*Flavobacterium* can synthesize xanthophyll, particularly the pigment zeaxanthin, which has significant economic value in nutrition and pharmaceuticals. Recently, the use of carotenoid biosynthesis by bacteria and yeast fermentation technology has shown to be very efficient and offers significant advantages in large-scale production, cost-effectiveness, and safety. In the present study, JSWR-1 strain capable of producing xanthophyll pigment was isolated from a freshwater reservoir in Wanju-gun, Republic of Korea. Based on the morphological, physiological, and molecular characteristics, JSWR-1 classified as belonging to the *Flavobacterium* species. The bacterium is strictly aerobic, Gram-negative, rod-shaped, and psychrophilic. The completed genome sequence of the strain *Flavobacterium* sp. JSWR-1 is predicted to be a single circular 3,425,829-bp chromosome with a G+C content of 35.2% and 2,941 protein-coding genes. The optimization of carotenoid production was achieved by small-scale cultivation, resulting in zeaxanthin being identified as the predominant carotenoid pigment. The enhancement of zeaxanthin biosynthesis by applying different light-irradiation, variations in pH and temperature, and adding carbon and nitrogen supplies to the growth medium. A significant increase in intracellular zeaxanthin concentrations was also recorded during fed-batch fermentation achieving a maximum of 16.69 ± 0.71 mg/l, corresponding to a product yield of 4.05 ± 0.15 mg zeaxanthin per gram cell dry weight. Batch and fed-batch culture extracts exhibit significant antioxidant activity. The results demonstrated that the JSWR-1 strain can potentially serve as a source for zeaxanthin biosynthesis.

## Introduction

Carotenoids are lipid-soluble pigments in different fruits, plants, fungi, microorganisms, and vertebrates. They are environmentally safe and possess therapeutic properties such as antioxidant, antimicrobial, and anticancer properties [1-3]. Recently, a growing interest has been in utilizing biological agents to produce colorants derived from plants and microorganisms. These natural colorants provide alternatives to synthetic dyes and pigments that are non-toxic, non-carcinogenic, and biodegradable. Pigments derived from microorganisms are favored due to their inherent stability and year-round cultivation potential [4]. Bacteria have great potential for producing bioproducts, such as pigments. In recent years, the production of bacterial pigments has become increasingly important as researchers have focused on identifying cost-effective and suitable cultivation media [5-9]. Bacteria species exhibit redundancy in synthesizing certain carotenoid pigments, a phenomenon associated with genes involved in carotenoid biosynthesis. Several research has shown that manipulating metabolic pathways linked to specific carotenoid synthesis can substantially improve production [10, 11]. It indicates that certain bacterial species are potential hosts for the biosynthesis of carotenoids [12]. Increasing the activity of key genes for making carotenoids has been the main focus of metabolic engineering of carotenoids in bacteria. The overexpression of these genes has been shown to increase carotenoid production [13, 14]. In addition, prokaryotic bacteria make them easier to adapt to genetic modification than eukaryotic algae and plant systems. However, additional efforts, including several strategies, will be necessary to enhance carotenoid biosynthesis in bacteria. Different strategies like selecting overproducing strains, controlling their culture conditions, stress, and metabolic engineering tools improved the production of carotenoid pigments on the industrial scale. There is a significant amount of unexplored microbial biodiversity across many ecosystems that can be used in carotenoid manufacturing processes.

Furthermore, microorganisms have been observed to accumulate carotenoids in response to biotic and abiotic stressors by upregulating the carotenoid metabolic pathway. Several bacteria demonstrate certain ideal stress conditions that enhance the production of beneficial metabolites [2, 15, 16]. The use of stress-induced methodologies has shown to be very effective in improving the biosynthesis of carotenoids in bacterial species. However, it is essential to recognize that such a system can inhibit cell growth and trigger oxidative damage. Accumulating stress-induced carotenoids is advantageous for synthesizing the required carotenoids [17]. Previous studies have shown the impact of temperature, salinity, light, and pH on some bacterial species' carotenogenesis and related metabolic pathways [18-20]. The main objective of the current investigation is to examine the prevalence of xanthophyll pigment overproduction in a newly identified bacterial species. Notably, the strain produced maximum amount of zeaxanthin in fed-batch fermentation. Zeaxanthin, a hydrophobic carotenoid with a yellow color, finds use as a pigment in several food and cosmetic formulations. Also, zeaxanthin play a significant role in developing visually appealing yellow pigmentation in the meat, skin, and eggs of chickens and fish [1, 21]. Additionally, zeaxanthin has potential in preventing age-related macular degeneration, thereby positioning them as a prospective candidate for use within the pharmaceutical industry [22]. Zeaxanthin present in plants, algae, and photosynthetic bacteria, mainly in mono- and diglycosides [23]. It is an auxiliary pigment for light absorption and protects against UV radiation and oxygen radicals. Several microorganisms have been found to synthesize zeaxanthin, such as *Synechocystis* sp., *Paracoccus zeaxanthinifaciens*, *Muricauda lutaonensis*, *Flavobacterium* species and *Yarrowia lipolytica* [24-28]. Among them, *Flavobacterium* species are potential candidate for zeaxanthin over-production in industrial scale. The Flavobacteriaceae family, which belongs to the Bacteroidetes phylum, consists of Gram-negative bacteria with a road-shaped morphology, displaying either motile or nonmotile characteristics. The examined taxonomic group encompasses an average of 130 district species [29]. The group encompasses many taxonomic groups found in marine and Antarctic environments, characterized by their ability to survive in cold temperatures (psychrophilic) or tolerate such conditions (psychrotolerant) and their need for sodium ions for growth and development [30-33]. *Flavobacterium* sp. can produce xanthophyll, particularly zeaxanthin, which constitutes around 95% to 99% of the carotenoid composition [34]. In the present study, the experimental strain JSWR-1 isolated from reservoir. The morphology of the strain, optimal growth parameters, C/N source on different conditions, pigment intensity, and duration of pigment production were compared. Batch fermentation effectively produced xanthophyll pigments under the optimized experimental conditions. During batch fermentation, different light stress conditions were used. This study was an initial approach to identifying the interaction of variables, the influence of pH, and temperature control strategy. Notably highest xanthophyll pigment production was achieved by fed-batch fermentation at 120 h incubation. After that, additional efforts will be made to improve the cultivation conditions and to enhance the production in a further scale‐up.

## Materials and Methods

### Microorganisms and Pigment-Forming Isolates Identification

**Fig. 1:**
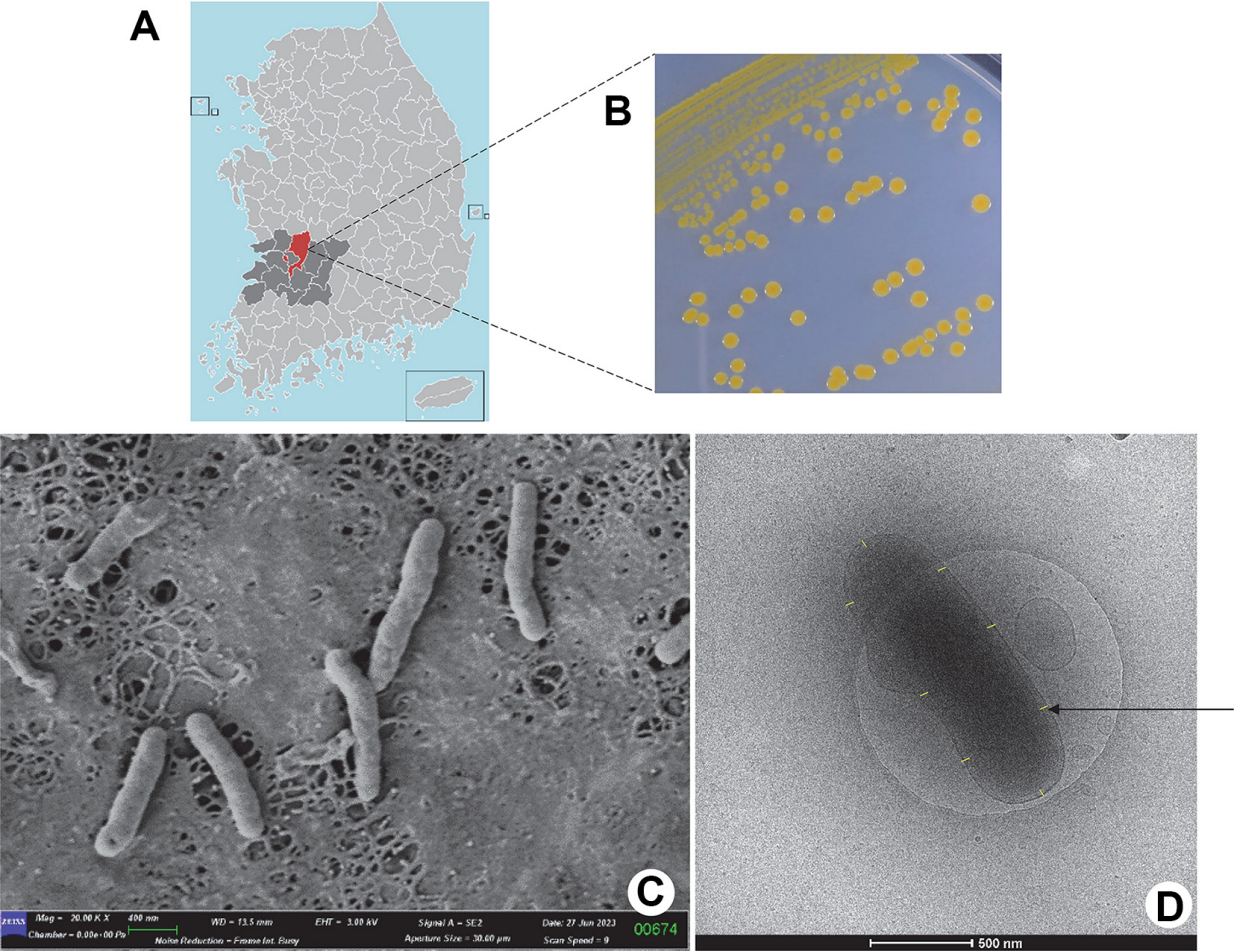
Isolation and identification of *Flavobacterium* species JSWR-1. (**A**) A red-marked region on the map indicates Wanju-gun, South Korea (Source from Wikipedia); (**B**) Isolates JSWR-1 showed colony morphology on R2A agar and pigmentation. (**C**) JSWR-1 displayed road-shaped colony morphology on scanning electron microscopy (SEM, x 10,000). (c) TEM images of JSWR-1, the arrow indicated the region of zeaxanthin accumulation.

Water samples were obtained from a freshwater reservoir in Wanju-gun, Republic of Korea (35° 49' 32" N and 127° 02' 28" E; Fig. 1A) and prepared for plating on R2A agar (Difco, USA). Subsequently, the plated samples were incubated under dark conditions at 28 ± 1°C for 7 to 12 days. Pure colonies of the strain were produced in a subsequent cycle of quadrant streaking on sterile agar plates containing 1.5% (w/v) agar. The stock cultures were preserved with 20 glycerol and kept at -80ºC. Five bacterial isolates exhibit the characteristic of producing a yellow pigment. After obtained the xanthophyll pigmentation JSWR-1 isolates, Gram staining was performed using an optical microscope (Leica DM2500, Germany). The morphology of bacterial cells was analyzed using scanning electron microscopy (Carl Zeiss Gemini SEM 300) and transmission electron microscopy (Cryo-TEM, 120 kV Talos L120C, FEI, Czech).

### Complete Genome Sequence of JSWR-1

Complete genome assembly, 16S rRNA gene sequence, and additional genomes were discovered using Macrogen (PacBio RSII; Illumina NovaSeq, Republic of Korea). As part of the strain JSWR-1 genome assembly, HiFi (high-fidelity) reads are used to make high-quality de novo assemblies of microbial genomes. Error correction was conducted with Pilin (v1.21). The 16 rRNA gene sequence of JSWR-1 was identified by pairwise comparison using the EzBioCloud database [35]. In addition, MEGA software application (version 7.0.26) was used to construct the phylogenetic relationship between 18 closely related strains of *Flavobacterium* species. The genomic sequences of the strain have been deposited in the GenBank database with the accession number CP132329.

### Inoculum and Culture Conditions

The bacterial inocula was cultured in R2A broth for 48 h at 28ºC with agitation at 120 rpm. The growth of individual colony cultures was successful, resulting in a culture density of OD ± 0.990 (OD_600_) [36]. A specific volume of inoculum (v/v) derived from an exponential phase culture is introduced into the sterilized R2A liquid medium. The batch-fermentations were conducted using 100 ml Erlenmeyer flasks containing 50 ml of culture media. To investigate the impact of temperature on pigment formation, the incubation temperature was manipulated to various levels ranging from 10 to 40°C. The study aimed to assess the isolated strains' psychrotolerant qualities and carotenoid-production capabilities. The temperature has been improved to facilitate further pH and C/N ratio tuning. To assess the impact of pH on pigment synthesis, the pH levels of the culture medium were modified to include a range of values from 5 to 11, including intermediate values such as 5.5, 6, 6.5, 7, 7.5, 8, 8.5, 9, 9.5, and 10 (Table S1). Glucose and yeast extract used as carbon and nitrogen sources in the experiment. Further, light-induced study was carried out in optimized culture condition (10°C, pH 8.5, C: N is glucose 10 g l^-1^ and yeast extract 5 g l^-1^). The culture was subjected continuous irradiance by light emitting diodes with four different combinations of monochromatic narrowband light red, green, blue, and white light (RGB 12 V, MANI LED, South Korea). Linear LED lights were placed on the bottom side of the culture flask in each experiment. Light intensity was measured using digital light meter TES 1337 (TES Electrical Electronic Corp., Taiwan). The study measured growth, biomass production, carotenoid content, and concentration. During incubation, samples were collected periodically at 24 h intervals after 48 h.

### Fermentation Conditions

JSWR-1 was pre-culture in R2A broth and transferred into broth medium supplemented with glucose, yeast extract and MgSO_4_ media comprised 10 g l^-1^ glucose, 5 g l^-1^ yeast extract, 0.7 g l^-1^ MgSO_4_. The initial pH of media was adjusted as pH 8.5 before sterilization. Two different volumes of batch and fed-batch fermentation were carried out. Batch fermentation were performed in 50 and 300 ml medium in 100 and 1 L baffled Erlenmeyer flask and incubated at 10°C on rotary shaker at 120 rpm, for 144 h with continuous white light exposure. The fed-batch fermenter operating volume is 2.5 L in a 5 L container at 10ºC (Marado-PDA, CNS Co., Ltd., Korea). To initiate fermentation, 25 ml of seed culture is inoculated aseptically into the fermenter. The pH of the fed-batch culture media was autonomously adjusted to pH 8.5 utilizing 1 N KOH/HCL. The agitator speed was 200 rpm, and a sterile air supply was initiated with a continuous mass flow controller dissolved oxygen to meet the strain requirement. If excessive foaming was detected, antifoam 204 (Sigma, USA) agent was added manually. A feeding strategy was implemented in order to attain high cell densities with a more conservative feeding method. The supplementation included 700 g/l glucose and 8 g/l magnesium sulfate. A personal computer controlled the system during fermentation, and a screen displayed the conditions of the culture. At 48, 72, 96, 120, and 144-h intervals, samples were collected for analysis of dissolved oxygen, pH, and temperature.

### Xanthophyll Pigment Extraction

One microliter of the fermented media (BC and FBC) were used to quantify yellow carotenoid production, and each samples were centrifuged at 4°C for 20 min at 10,000 rpm to recover the cells by decanting the supernatant. For dried biomass yield, 50 ml of medium was used for cell-down, and the resulting biomass was stored at -80°C (PHC Corp., Japan) for 48 h before lyophilization [37]. The results were calculated in dry-weight micrograms per liter of culture medium (mg/l). Total carotenoid was extracted from recovered cells using acetone at 55°C and ultrasonic bath (220 V and 60 Hz, JINWOO JAC Ultrasonic device, Republic of Korea) until cells were completely bleached. The xanthophyll pigment was separated by centrifugation at 10,000 rpm for 20 min at 4°C, and it was confirmed that no pigments remained in the cell fragments after extraction. Deep yellow-orange colored supernatant and colorless pellet were yielded. This colored supernatant was filtered through a 0.2 μm nylon syringe filter (HENKE-JECT, Korea) and air-dried in a dust-free N_2_ flesh (TLS Technology, TLS HPS-1, Republic of Korea). The dried samples were dissolved in a known quantity of methanol following low-light tests. The extract solutions UV/visible spectra were recorded at 230-700 nm using spectrophotometer (TECAN infinity M200 plate reader, Switzerland). The total carotenoid content (TCC) was determined by measuring the optical density of the sample in 480 nm [38].

### Xanthophyll Pigment Quantification

The total carotenoid content (TCC) was determined using the procedures described by Zhao *et al*. 2019 and Liu *et al*. 2020 [39, 40]. The carotenoid concentration in a batch and fed-batch fermented medium were quantified using high-performance liquid chromatography (HPLC, Agilent, USA), equipped with a UV-visible photodiode detector. At a wavelength of 470 nm, the analysis was conducted on a 250 × 4.6 mm. D. S-3 m YMC carotenoid column. A solvent mixture of ethyl acetate, methanol (95:5) ammonium acetate (0.4 g/l), acetic acid (0.1%) in methanol with six different mobile phases. The flow rate was fixed as 0.6 ml/min. Between 300 nm and 700 nm, carotenoid 0.78-100 μg/ml detection was performed using DAD (Diode array detector). The retention time (t_R_) of specific carotenoids on a HPLC calculated by standard retention time and linear relationship. Four analytical standards of zeaxanthin, β-carotene, and canthaxanthin (Sigma-Aldrich, Germany) were dissolved in methanol at a concentration of 0.78–100 g/ml, and a standard calibration curve was constructed for quantification.

### Xanthophyll Pigment Characterization

To confirm carotenoids content in FBC and BC extracts, TLC was used. FBC and BC extracts was spotted on HPTLC Silica gel 60 F_254_ plate (Merck, Germany). The plates were developed using petroleum ether, ethyl acetate 6:4 (v/v) was used for separation. Extracts were compared with standards: (1/10 mg/ml, zeaxanthin, lutein and β-carotene) from Sigma-Aldrich. The developing distance of 7.5 cm was reached in 14 min at room temperature (26°C). The plates were desiccated with a hair dryer and cool air discharge. Individuals were identified using pigment's R*_f_* [41, 42]. The type of carotenoid produced by JSWR-1 was identified and characterized by liquid chromatography coupled with MS. FBC and BC sample was prepared by extraction of the JSWR-1 lyophilized cells with acetone and undergone water bath sonication. Further samples were resuspended in methanol and examined in a LC system (ACQUITY I-Class plus FTN) and coupled to the electron spray-ion trap mass spectrometer XEVO G2-XS QTof (Waters Corp., USA). Analysis according to suggested protocol provided by Chungbuk National University, South Korea. For chromatographic separation, a BEH C18(1.7 mm, 2.1 × 150 mm) was used. The separation of 5 μl of bot BC and FBC extracts was performed on LC system. LC analysis was performed using BEH C18(1.7 mm, 2.1 × 150 mm) by isocratic elution with a 0.4 ml/min flow rate. The mobile phase A: 0.4 g/l ammonium acetate, 0.1% acetic acid in methanol and B: 0.4 g/l ammonium acetate, 0.1% acetic acid in ethyl acetate: methanol (95:5). The column temperature maintained at 40°C, and UV detection was performed at 450 nm. The mass spectra were recorded in the positive ions mode in the mass range from 100 to 1500 *m/z*. The zeaxanthin was identified using the standard (14681-1MG-F, Sigma-Aldrich). The extraction of total fatty acids was performed on 100 mg of dry biomass pellets using a solvent-based approach. The biomass was homogenized after adding 3 ml of extraction solvent. During the second step, a volume of 0.5 ml of an aqueous buffer was introduced into the system. The mixture was then subjected to vortexing and afterward filtered using a syringe filter with a plunger attached. The lipid extract underwent transesterification and was evaluated using GC-FID (Agilent 7890A). The methodology used in the fatty acid extraction kit (Sigma) manufacturing procedures was followed.

### In vitro Antioxidant Activity of Yellow Carotenoids

The ability of the test samples to scavenge free radicals was evaluated according to Xiao *et al*. 2020 [43]. One microliter aliquot of DPPH solution was mixed with 800 μl of Tris-HCl buffer (pH 7.4) in a test tube BC and FBC. Then, 200 μl of the testing sample solution was quickly added and thoroughly mixed with a 0.1 to 4 mg/ml concentration. The solution was incubated for 30 min at RT (25°C). The solution's absorbance at 517 nm was measured. The blank solution contained 1,200 μl of ethanol and 800 μl of a pH 7.4 Tris-HCl buffer. The inhibition ratio (%) was calculated using the following formula: Inhibition ratio (%) = (A1 - A2) × 100/A1, where A1 is the absorbance of the addition of ethanol instead testing sample and A2 is the absorbance of the testing sample solution. The IC_50_ value of the extracts was determined by linear regression analysis of the graph of the average percentage of DPPH inhibitory activity, measured in triplicate, compared to the equivalent concentrations of the tested samples. The ferric reducing antioxidant power (FRAP) assay was performed using a FRAP Assay Kit (Sigma) according to the manufacturer's instructions.

### Statistical Analysis

The experiments were performed in triplicate to determine the mean values and standard deviations. Duncan's multiple range tests (DMRT) were used for conducting pairwise or individual (one-to-one) comparisons in the analysis of variance (ANOVA). A significance level of *p* < 0.05 was used to determine the individual differences. The statistical analyses were conducted using the IBM SPPS statistical program (Version 27, SPPS Inc., USA).

## Results

### The Isolation and Identification of *Xanthophyll* Pigment Producing Bacteria

A total of five gram-negative *Flavobacterium* species with yellow pigmented strains were isolated from water samples as pure cultures. Among the isolates JSWR-1 exhibited notable characteristics, such as fast growth and a high pigment production capacity. Therefore, this strain was selected for further optimization for over-production of carotenoid. On a R2A agar plate containing the isolates JSWR-1, circular with a thin spreading edge, and orange-yellow colonies were observed (Fig. 1B). The electron microscopy images exhibit road-shaped colonies, the cells were 1.30-1.50 μm × 0.5-0.6 μm in size (Fig. 1C and 1D). The sequenced of the JSWR-1 whole genome was performed using PacBio SequelII system, Illumina platform by Macrogen Inc. [44].

### Complete Genome Sequence of JSWR-1

The genome was a circular chromosome with 3,425,829 base pairs and a G+C content of 35.2%. The genome sequence analysis identified 2,941 protein-coding sequences, of which 2,770 were annotated with the EggNOG database (Table 1). This investigation involved a comprehensive annotation of twenty-four distinct categories. The functions included metabolism, nucleotide transport, coenzyme transport, lipid transport, inorganic ion transport, biosynthesis of secondary metabolites, transport and catabolism, replication, recombination, and repair (Fig. S1). The genome also includes 62 transfer RNA-encoding genes and 15 ribosomal RNA-encoding genes. The 16S rRNA gene sequence of JSWR-1 was identified by pairwise comparison. *Flavobacterium* erciyesense and *F. turcicum* 16S rRNA sequences were the most similar, with a similarity of 99.64% and 98.95%, respectively. The MEGA software application (version 7.0.26) was used to construct the phylogenetic relationship between 18 closely related strains of *Flavobacterium* species. The JSWR-1 strain exhibits two distinct branches within the *Flavobacterium* genus, strongly supported by significant bootstrap values (Fig. S2). These phylogenetic tree branches belong to a clade consisting exclusively of recently described *Flavobacterium* species, including *F. erciyesense*, *F. turcicum*, *F. collinsii*, *F. aquiphilum*, *F. gilvum* and *F. chungangense*. JSWR-1 genome contained carotenoid biosynthesis-related *crt* genes. The genome sequence has been deposited at Gene bank, the accession number CP132329 (*Flavobacterium* sp. JSWR-1). In addition, culture has been deposited in Korean Agricultural Culture Collection (KACC) with the accession number KACC 81264BP.

### Effect of Temperature

To determine the impact of different temperature on biomass and total carotenoid production, the culture cultivated in minimal media. The batch cultivation results at nine different temperature range from 10-40°C indicate that the different growth rate, biomass and zeaxanthin yield. The strain JSWR-1 was cultivated in aerobic condition and incubated two different time intervals (48 and 72 h) under dark. Maximum growth rate and carotenoid concentration was recorded at 10°C. The maximum dry biomass yielded was observed at 25°C (860 mg/l) and minimum at 30°C (400 mg/l). There was growth and biomass yield were observed at 35 to 40°C. Notably at the end of 72 h incubation, the zeaxanthin concentration significantly higher at 10°C (3.46 ± 0.09 mg/l) than compared to 15 and 20°C (Fig. S3A-S3C).

### Effect of pH on Zeaxanthin Production

**Fig. 2:**
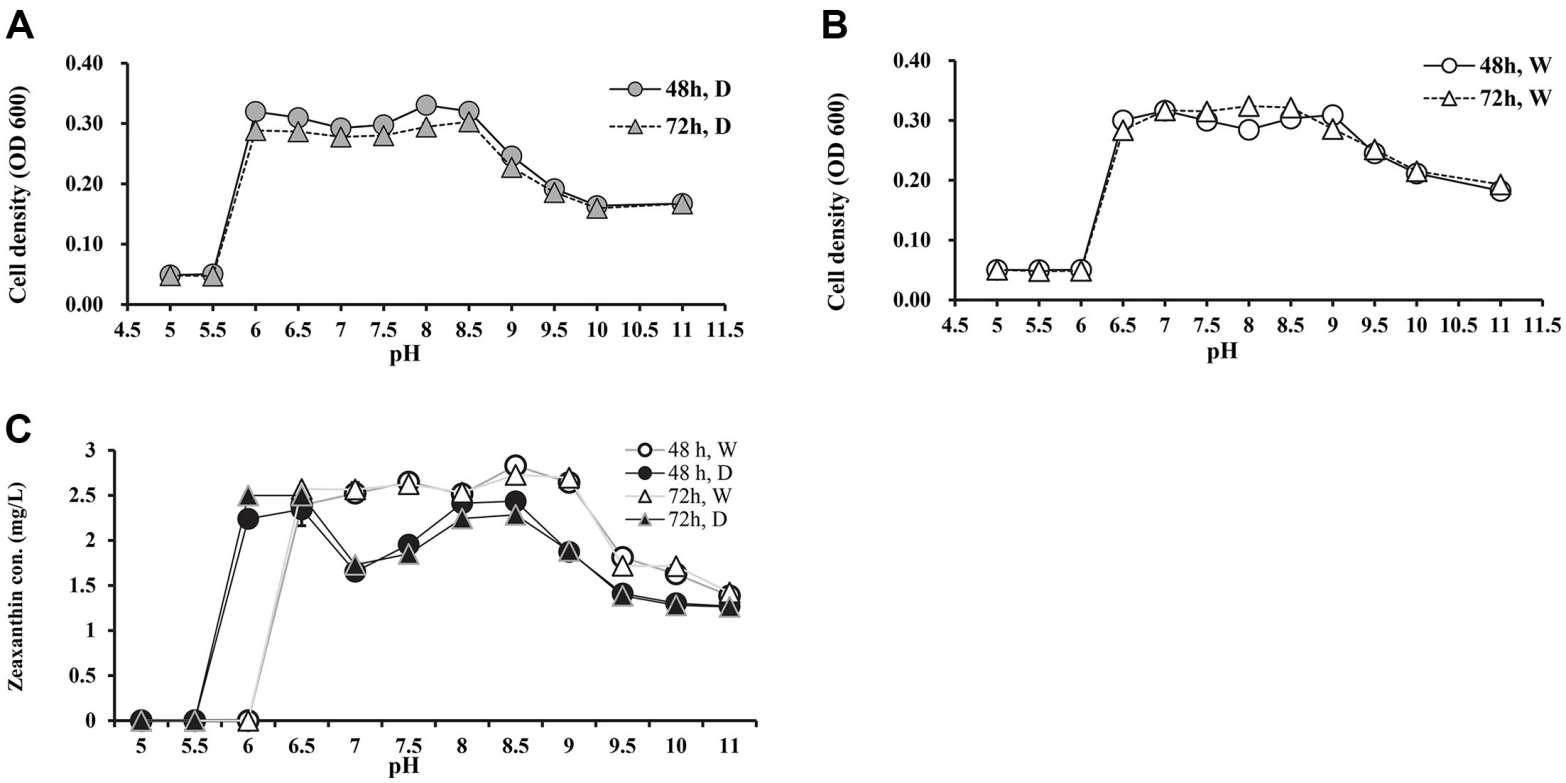
Effect of pH on growth and zeaxanthin production of JSWR-1. (**A**) Specific growth in two different time intervals at dark incubation. (**B**) Specific growth in two time intervals at white illumination. (**C**) Zeaxanthin production on different pH and incubation conditions.

To determine the optimal pH, JSWR-1 was cultivated at pH range between 5 to 11 in 100 ml baffled flask. The pH had significant influence the growth and total carotenoid production. As shown in Fig. 2, the initial pH significantly influenced the growth and zeaxanthin production from JSWR-1. Specific growth and zeaxanthin production rates increased when the pH 8.5, while they decreased at high and low pH (Fig. 2A and 2B). The highest zeaxanthin concentration of 2.83, 2.73 mg/l was yielded at 48 and 72 h in white illumination (Fig. 2C). The maximum biomass yield was observed at pH 8 (525 mg/l) and 8.5 (590 mg/l) in dark incubated culture. In contrast, the negligible growth, absence of biomass and zeaxanthin production were observed at pH 6, 5.5 and 5. White illumination highly influence in bacterial growth and zeaxanthin production, while biomass yield was not significantly higher than dark incubation (Fig. S4).

### Effect of Carbon and Nitrogen (C/N) Concentration

**Fig. 3:**
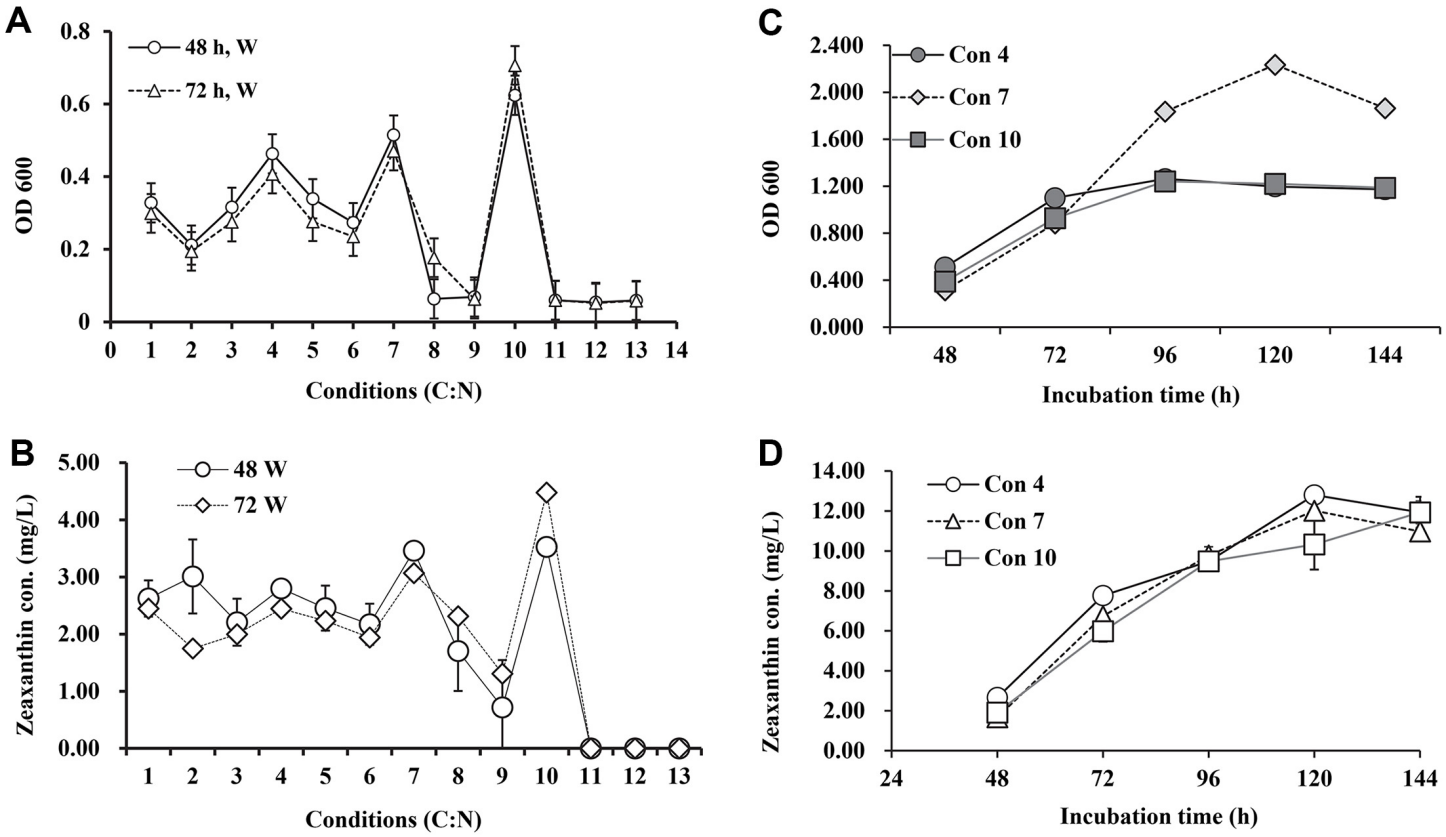
Effect of C: N ration on bacterial growth and zeaxanthin production of JSWR-1. (**A**) Specific growth in two different time intervals at white illumination. (**B**) Production of zeaxanthin from different C: N ratios. (**C**) Bacterial growth on selected C: N concentrations from different time intervals. (**D**) Maximum zeaxanthin production from selected conditions.

Bacterial growth and zeaxanthin production by JSWR-1 strain in different carbon and nitrogen (C: N) ratios are shown in the Fig. 3A & 3B. In minimal media both independent conditions and their interaction, significantly influenced the cell density and zeaxanthin production at pH 8.5. Conditions 10, 7, and 4 shown high cell density on white light exposure at 48 and 72 h incubation (Fig. 3A). In addition, zeaxanthin concentration was recorded maximum at 72 h incubation (Fig. 3B). The yield was significantly increased when comparted to control (2.34 mg/l). C: N ratio a further increased to 11 to 13 showed no positive effect on the bacterial growth (Fig. 3B). Glucose concentration 10 to 20 mg/l and yeast extract 5 mg/l dramatically influence on the growth and zeaxanthin production. Hence, to increase glucose concentration to 10 to 20 mg/l which significantly influenced on specific growth and zeaxanthin concentration, the spike depicted in Fig. 3A is clearly evident. Treatment condition 10 reached maximum zeaxanthin production (4.48 mg/l) at 72 h incubation, white illumination (Fig. 3B). Furthermore, condition 7 exhibit maximum cell density (OD_600_ 2.235 ± 0.01) at 120 h and zeaxanthin production reached maximum in condition 4 at 120 h (12.80 ± 0.01 mg/l) incubation (Fig. 3C and 3D). Besides, condition 7 also influenced the zeaxanthin production (12.02 ± 0.01 mg/l) at 120 h (Fig. 3D).

### Effect of Light Sources on Zeaxanthin Production

Light-induced carotenogenesis was carried out in different time intervals with optimized culture conditions. The culture grew in red light very intensively (OD_600_ 1.36) at 96 h, and maximum biomass dw (3.32 ± 0.05 mg/l) was recorded at 144 h. The average cell biomass ranges from descending order follows dark incubation (1 ± 0.3 to 2.74 ± 0.5 mg/l), white (0.29 ± 0.03 to 2.59 ± 0.13 mg/l), green (0.5 ± 0.3 to 2.45 ± 0.17), and blue (0.56 ± 0.02 to 2.3± 0.09 mg/l), respectively. Bacterial growth was initiated at 48 h and the maximum growth was recorded after 72 incubations. When white light exposure the strain JSWR-1 accrued 563.83 ± 32.75 μg/g DW cells of TCC at 72 h. In addition, the primary pigment zeaxanthin obtained maximum from white illumination 443 ± 0.08 μg/g and 12.80 ± 0.02 mg/l (Table 2). The minimum zeaxanthin concentration was observed in red light range between 3.51± 0.1 to 7.84 ± 0.07 mg/l. In addition, very minimum TCC (298.59 ± 43.84 μg/g) and zeaxanthin production was recorded in green light exposure (145 μg/g and 1.90 mg/l) Table 2.

### Over-Production of Zeaxanthin in Fed-Batch Fermentation

**Fig. 4:**
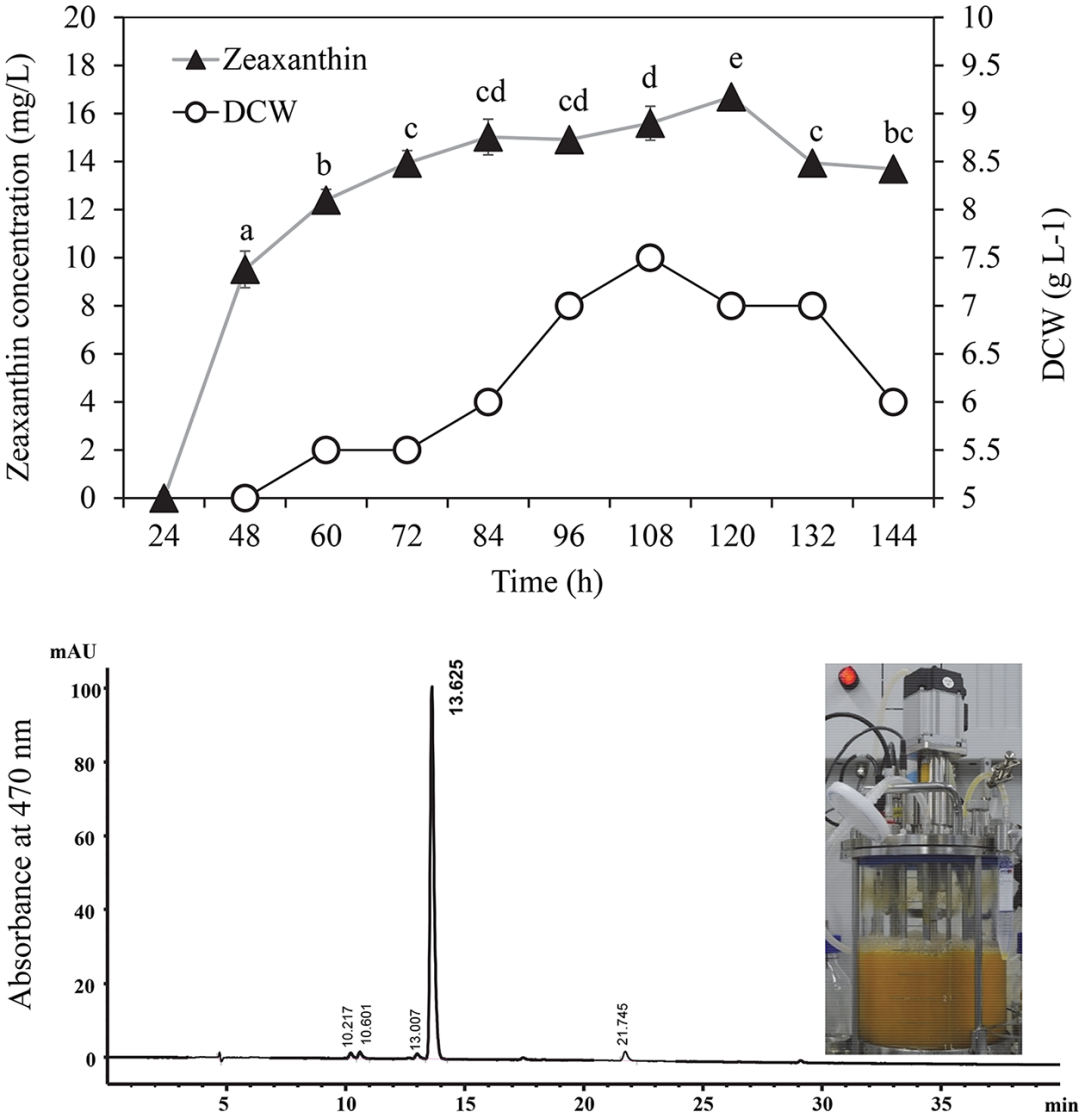
Fed-batch fermentation of *Flavobacterium* sp. JSWR-1. Glucose (10 g l^-1^) and yeast extract (5 g l^-1^). Zeaxanthin concentration represented in mean value ± SD (*n* = 2) respectively, different alphabetic letters indicate significant difference *p* < 0.05 by Duncan's multiple range test. Dry weight of the cell pellet was determined by difference after freeze drying (g l^-1^). The fed-batch fermenter vessel picture was capture at 120 h.

The optimized culture conditions significantly improved the production of zeaxanthin in batch-fermentation. The same culture conditions deployed in fed-batch fermentation to increase productivity further. High cell density was observed in fed-batch culture, and the cell growth was examined in a media containing 10 g/l of glucose and 5 g/l of yeast extract. The initial glucose concentration significantly influences on the cell growth. Controlled sterilized air flow (PV 5 L/min) influence cell growth, biomass and carotenoid over-production. by improving transfer of oxygen and nutrition to the JSWR-1 strain. The feeding was stope after 96 h, to allowed to enter a stationary phase. The maximum bacterial dry biomass (7.5 g/l) and zeaxanthin production (16.69 ± 0.71 mg/l) was recorded at 108 h and 120 h, respectively. Biomass and zeaxanthin production in fed-batch fermentation exhibited statistically significant time intervals difference (Fig. 4).

### UV-vis and HPLC Profile of Yellow Carotenoid from JSWR-1

**Fig. 5:**
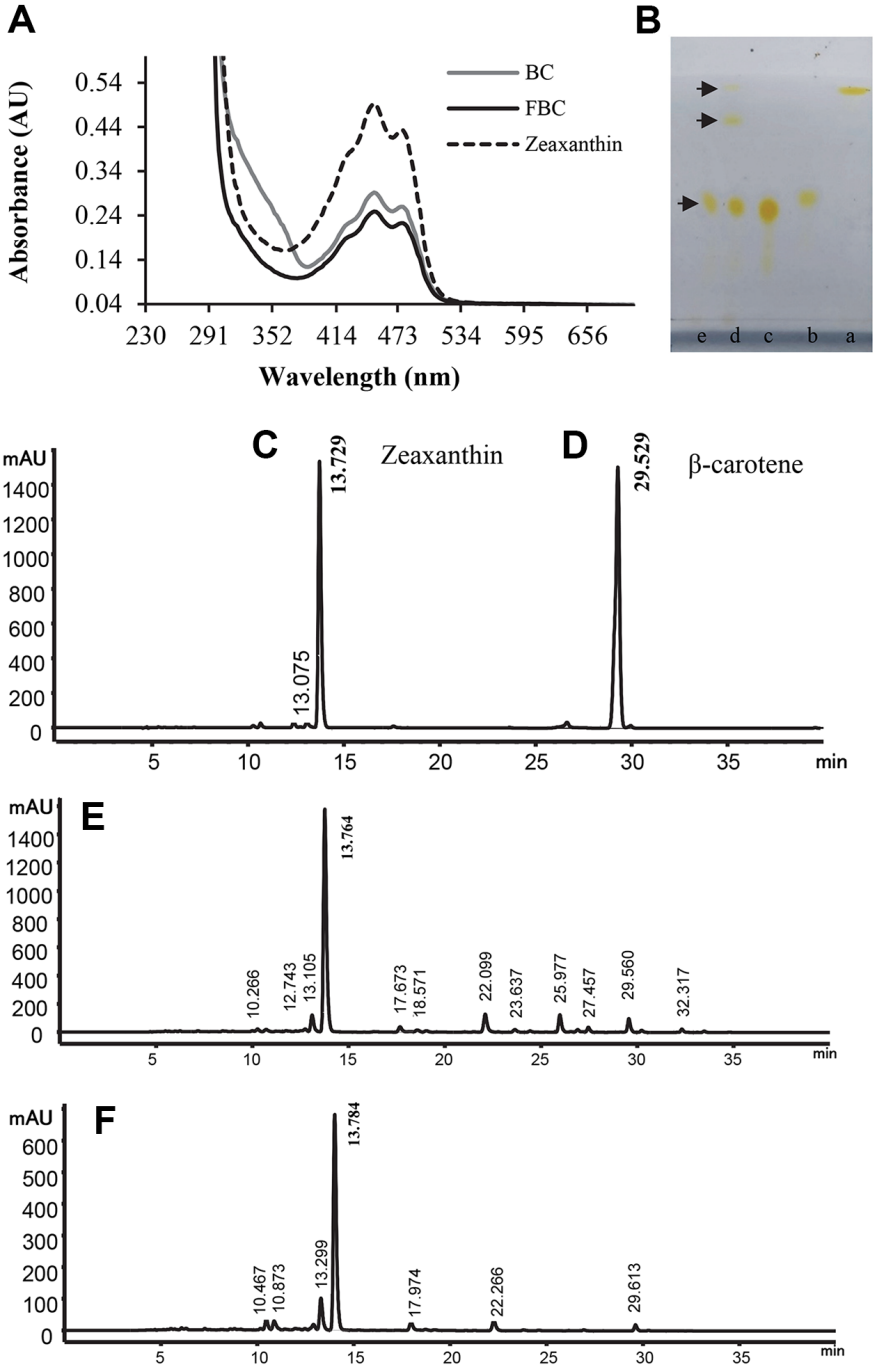
Chromatogram of yellow carotenoids from JSWR-1. (**A**) UV-VIS spectra of samples compared with zeaxanthin standard (dashed line), absorbance maximum of 450, 478 nm (**B**) Thin-layer chromatography of standard and samples (a, β-carotene; b, lutein; c, zeaxanthin; d, BC; e, FBC), the dashed arrow indicates the BC and FBC spots on TLC; (**C**) HPLC chromatogram of standards zeaxanthin, the retention time 13.729 min.; (**D**) HPLC chromatogram of β-carotene, the retention time 29.529 min.; (**E** & **F**) HPLC profile of BC and FBC.

FBC and BC extracts were analyzed qualitative and quantitative using UV-visible, TLC and HPLC. Zeaxanthin UV-Vis absorption maxima were measured in the visible range (450–500 nm) and correspond to xanthophyll pigment (Fig. 5A). FBC and BC exhibited maximum absorbance peaks at 450, 478 nm, these peeks were found to be identical to the peak observed in the standard (Fig. 5A). The minimal UV absorbance was recorded at 428 nm. In TLC profiling the authentic standards (zeaxanthin, lutein, β-carotene) R*_f_* values and retention time were matched with samples. The R*_f_* values of the standards 0.47, 0.52, 0.95, respectively. BC extract exhibit three yellow spots on TLC, and measured the R*_f_* value 0.48, 0.81 and 0.95. FBC extract show single spot, the R*_f_* value 0.48. The values 0.48 was similar with zeaxanthin, and both extracts R*_f_* values and spot color was identical to the standard zeaxanthin. In addition, BC R*_f_* value 0.95 identical to β-carotene (Fig. 5B). On HPLC two major peaks were observed with retention times 13.7 and 29.5 min, as deciphered by comparison with standards and no significant difference were observed. Although several other compounds eluted before and after the zeaxanthin (Fig. 5C-5F).

### LC-MS/MS and Fatty Acid Analysis

Xanthophyll pigment fragmentation patterns are identified by LC-MS/MS and obtained structural information about the specific carotenoids. FBC and BC extracts containing high intensity of zeaxanthin and other specific carotenoids based in the detection of unique fragment ions (Fig. 5A and 5B). The fragment *m/z* 569.434 similar to the reference standard and the molecular formula C_40_H_57_O_2_ (Fig. S5). The fragmentation spectrum of FBC showed a low intensity signal m/z 475.30, m/z 429.24, m/z 368.43, and m/z 338, was observed as the most abundant product ion formed from the molecular ion of *m/z* 569 for zeaxanthin. Other carotenoid formed minor compounds. Extracted fatty acid from FBC culture was derivatized to fatty acid methyl ester (FAMEs) subjected to GC analysis (Fig. S6). The results showed that the JSWR-1 FBC produced maximum saturated fatty acid namely C15:0, C15:1, C16:0, C16:1 and C18:0 were detected. In addition, monounsaturated fatty acid erucic acid (C22:1n9) accounting for a percentage of 0.803% of the total fatty acids, while the unsaturated fatty acids accounted for 99.20% of the total fatty acids. The two major unsaturated fatty acids were recoded as C16:1 (50.06%) and C15:0 20.58%.

### In Vitro Antioxidant Activity of *Xanthophyll* Pigments from Batch and Fed-Batch Fermentation

**Fig. 7:**
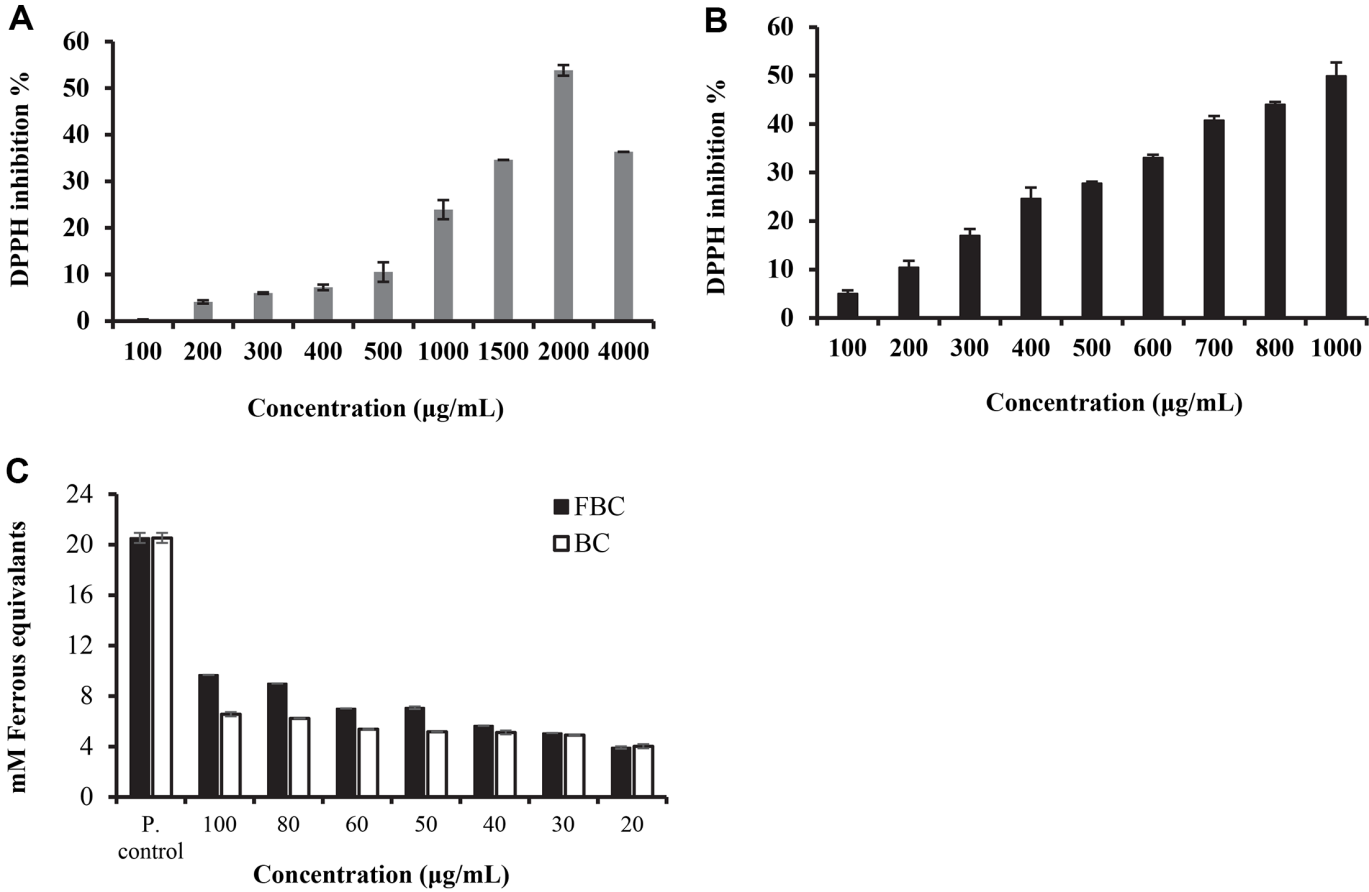
Antioxidant activity of FBC and BC from JSWR-1. (**A** & **B**) Radical scavenging activity in the DPPH assay of BC and FBC extracts; (**C**) Antioxidant capacity was measured using the FRAP assay. Error bars represent ± SD of the mean value, *n* = 3 independent experiment in which each sample was analyzed in duplicate.

These results of DPPH and FRAP assays showed fed-batch culture acetone extract as the most active antioxidant of the JSWR-1 extracts investigated. The DPPH free radical scavenging capacity of FBC, BC and ascorbic acid evaluated with different concentration gradients. FBC and BC inhibition percentage range between 5.02 ± 0.67 to 49.91 ± 0.281 and 4.13 ± 0.35 to 53.83 ± 1.14, respectively (Fig. 7A and 7B). The commercial zeaxanthin and ascorbic acid shown high radical scavenging ability, IC_50_ value of zeaxanthin 82.74, and ascorbic acid 12.30 μg/ml (Fig. S7). The IC_50_ value of FBC and BC was observed as 0.933, 1.75 mg/ml, respectively. The FRAB ability of JSWR-1 extracts, a measure of the reducing power, sowed that FBC and PC produced a dosed dependent antioxidant effect. At 100 μg/ml, the mean FRA*P* values of FBC and BC was 9.68, 6.56 μM, respectively. The values are decreased significantly the concentration between 100–20 μg/ml (Fig. 7C).

## Discussion

*Flavobacterium* species are being investigated for their potential to produce zeaxanthin, a natural pigment with important commercial value [45]. To achieve this, it is necessary to use strains of better quality than the native strains. In addition, mass manufacturing necessitates the utilization of optimum culture conditions and the provision of adequate nutrients and suitable lighting conditions on a large scale. The main goal should be to decrease production costs and utilize economical nutrient sources to enhance the process. Researchers have suggested that certain types of bacteria, such as *Flavobacterium*, *Erwinia*, *Formosa*, and *Muricauda*, can produce zeaxanthin [23, 34, 46]. *Flavobacterium* strains obtained from various aquatic environments, such as the Antarctic and other glacial sites, have been recognized as potential candidates for producing zeaxanthin [47-50]. The study described the isolation of JSWR-1, which can produce zeaxanthin as the primary carotenoid. The study also discussed increasing zeaxanthin production by utilizing optimized growth conditions for industrial scale-up. The strain was isolated from a freshwater reservoir and more adapted to cold environments. The 16S rRNA sequence of the strain JSWR-1 showed the highest level of identity (99.64%) with the strain *F. erciyesense* (F-328)^T^. The identified strain *Flavobacterium* sp. JSWR-1 exhibited yellow pigmentation in maximum level in fed-batch fermentation. Consequently, there is a significant demand for a novel bacterial strain capable of producing zeaxanthin as a single pigment in large volumes, which could be a candidate for industrial-scale zeaxanthin production [51, 52]. Barauna *et al*. (2017) proposed that the association between xanthophyll pigments and cold adaptation may be attributable to the pigments ability to modulate membrane fluidity, protect against UV radiation, reduce oxidative stress and which is associated with photoprotection [53, 40, 47]. In the study JSWR-1 strain produced the highest amount of zeaxanthin at 10°C and 8.5 pH and no growth was observed above 35 degrees Celsius. *Flavobacterium* is common among cold-adapted species, especially psychrophilic ones [32]. Whereas carotenoids in bacterial cells protect them from UV radiation and oxidative stress and are involved in the mechanisms of membrane fluidity (damage) mechanisms, lycopene inhibits membrane fluidity [54, 55]. Correctly maintaining the fluidity and structure of the cell membrane is essential for growth at low temperatures and for regulating nutrient transport. Studies in vitro have shown that zeaxanthin, a polar carotenoid, confers greater membrane rigidity than non-polar carotene [56]. Notably, pH was the most influential parameter in growth, and cell wall formation in bacteria. In addition, the medium pH significantly affected carotenoid production, with optimal pigment production occurring at a neutral pH. At a pH of 6.9, *F. multivorum* produces a maximal carotene concentration of 7.7 g/ml [57]. In the study, the initial pH of 8.5 in batch fermentation significantly increased biomass yield, growth, and zeaxanthin production. Below pH 6.5, no growth or pigment production were observed. Hence, alkaline pH considerably influences the biosynthesis of carotenoids under stress conditions [58]. In addition, the optimized growth conditions of 10°C and pH 8.5 were used to increase the effect of the C/N ratio and light sources on zeaxanthin production. Sources of nitrogen and carbon are crucial for zeaxanthin production. The present study investigated the effect of various conditions of carbon and nitrogen sources on the growth and production of zeaxanthin by JSWR-1 in a minimal medium (R2A). The greatest growth was supported by 10 g/l of glucose and 5 g/l of yeast extract. Yeast extract was the primary nitrogen source for JSWR-1 growth and zeaxanthin production. A supplement containing a high concentration of yeast extract (more than 5 g/l) inhibited the production of pigments and affected bacterial growth. During fermentation in batches, JSWR-1 produces 12.5 mg/l of zeaxanthin and in fed-batch fermentation enhanced the yield to 16.69 mg/l. Alcantara and Sanchez (1999) demonstrated that yeast extract (1%) promoted maximal zeaxanthin production in *Flavobacterium* species [59]. The strain produced between 10 and 40 mg zeaxanthin per liter under optimal culture conditions and media containing glucose and maize steep liquor [60]. In addition to nitrogen deficiency and the absence of MgSO_4_, a high glucose concentration also inhibits pigment production and growth [59]. An increase in cell mass and zeaxanthin yield will result in a rise in total carotenoid production. Mata-Gomez *et al*. (2014) and Saejung and Apaiwong (2015) have previously reported the utility of culture broth supplementation with C/N sources, light, and low temperature as stimulants for carotenoid production in various microorganisms [18, 61]. The yield of zeaxanthin was increased by continuous supplementation with fatty acids, amino acids, and trace metal elements, most significantly at low temperatures [19]. Aeration and agitation serve a crucial role in pigment production during fermentation. Oxygen is required for the desaturation, cyclization, and oxygenation of carotenoids by *Flavobacterium* [62]. To enhance *Flavobacterium* zeaxanthin production, a fermenter with a high oxygen transfer rate is preferred [19].

Several conditions, including low temperature, alkaline pH, and exposure to various light sources, are favorable to zeaxanthin production in JSWR-1. In addition, five distinct light sources were employed; under all of these conditions, the induction of total and specific carotenoid accumulation is typically accompanied by a decrease in cell growth due to the light sources effect on metabolic function. However, red light inhibited zeaxanthin production, promoting cell proliferation and biomass production. While white, red, and blue light treatments significantly increased the total carotenoid content, The exposure of *Pseudomonas aeruginosa* to the colors red and blue increased cell proliferation, biomass, and extracellular pigmentation [63]. According to Sharma *et al*.(2020), the accumulation of fatty acids in the cells is triggered by red and white light shifting [64]. This article produces varying levels of carotenoids in response to various light sources (Table 2). JSWR-1 contains more carotenoids and produces more carotenoids in response to exposure to white light. The highest zeaxanthin production was observed under white illumination (12.80 ± 0.02 mg/ml and 443 ± 0.08 μg/g, dry weight). Numerous studies have demonstrated that pigment production is white light-dependent. Also, it was seen that certain carotenoid biosynthesis genes (*crt*) were controlled by light in non-phototrophic bacteria [65]. Even though the exact way that carotenoids are made is still unknown, finding and describing the specific binding protein that links with *crt* genes will help to understand the light-dependent promoters. As these light sources produce stress conditions for the strains and sequentially alter gene expression and the metabolic cycle, they are anticipated to stimulate zeaxanthin production. JSWR-1 genome annotation has revealed the presence of two genes involved in the biosynthesis of carotenoids. crtB, which encodes phytoene synthase with an EC number of 2.5.1.32, and crtN, which encodes phytoene biosynthesis desaturase with an EC number of 1.3.8.-, are the relevant genes. The crtK-2 gene encodes the TspO (tryptophan-rich sensor protein). Armstrong *et al*. (1989) found that TspO is a membrane protein that participates in the intact pathway for carotenoid biosynthesis [66]. Moreover, it is linked to bacterial mechanisms related to stress and disease. Enoyl-CoA hydratase, which is encoded by the *crt* gene (E.C. 4.2.1.150), is required for fatty acid metabolism and polyhydroxyalkanoate (PHA) synthesis [67]. The fatty acid methyl palmitate’s methyl ester also stimulates zeaxanthin production in *F. multivorum* and *Paracoccus zeaxanthinifaciens* [27, 68].

Members of this genus can be utilized for the production of zeaxanthin because they can produce large quantities of all-trans-zeaxanthin as their primary carotenoid. The JSWR-1 produced a maximal zeaxanthin concentration of 16.69 ± 0.71 mg/l from fed-batch fermentation culture. The feeding parameters were optimized based on the specific growth rate, and feeding was initiated once the pH level dropped below a certain threshold, using a predetermined initial specific growth rate. The feeding approach based on progressively declining typical growth rates was initiated when the biomass concentration reached an OD_600_ of 1.36. The previous study described the production of β-carotene using the fed-batch methodology and scale-up strategy [69]. In the study, we described the isolated *Flavobacterium* sp. JSWR-1, which is capable of producing zeaxanthin as the primary carotenoid. Media composition, physicochemical parameters such as temperature and pH, and process-specific conditions (agitation, aeration rate, and light sources) may influence carotenoid production during fermentation. All these parameters affect cellular proliferation and specific carotenoid production [65, 69]. Many researchers have used *F. multivorum* to produce excess carotenoids. Table 3 contains examples of bacterial strains that produce excessive amounts of zeaxanthin. *Flavobacterium multivorum* has attained its maximal production level [34, 70]. During fed-batch fermentation, the JSWR-1 strain produced excessively yellow pigmented cells and a 7-fold increase in zeaxanthin concentration. However, other native and recombinant bacterial species also produce zeaxanthin [71-75].

The UV-Vis absorbance peak exhibited typical spectral characteristics of xanthophyll pigmented producing bacterium *Erythrobacter* sp. SDW2 strain [77]. The LC-MS/MS analysis of the yellow carotenoid indicates that the main carotenoids synthesized by JSWR-1 are classified as zeaxanthin. Zeaxanthin esters were identified as the primary chemical produced. The xanthophyll pigments lutein and zeaxanthin differ in their structure, with lutein having fewer double bonds. Additionally, lutein has unique light-absorbing properties. Both carotenoids are structural isomers, and their distinctions cannot be detected using mass spectra. Zeaxanthin and other carotenoids are created in psychrophiles, as mentioned in functions [47, 58]. Therefore, zeaxanthin and lutein play a crucial role in preserving the structural integrity of cells in cold-adapted bacterial species. Based on the molecular mass (m/z) of 569.43, we have determined that the compound is either zeaxanthin or lutein. From this, we can conclude that JSWR-1 can produce both carotenoids, with zeaxanthin being the predominant one under the specific cultural circumstances studied. The study found that exposure to light and temperature can promote isomerization. However, this change was not detected by the mass spectral analysis. However, the absorbance spectra in the study revealed a peak at a shorter wavelength, indicating the presence of cis forms [20]. Nevertheless, the FBC and BC samples displayed the highest values for UV-Vis absorbance spectra at 450, 478 nm, and 428 nm, indicating the existence of zeaxanthin in its all-trans form in the extracts. The oxidized form of carotenoid is not detectable from 475 to 300 m/z in LC-MS/MS spectra. The high concentration of all-trans zeaxanthin in this strain may have developed as a defense mechanism against the adverse effects of light and temperature stress. This provides evidence that the pigments present in JSWR-1 can reduce cellular stress caused by low temperatures and light exposure. The strain generates a small quantity of lutein and β-carotene, and using metabolic engineering tools may increase the synthesis of these required carotenoids. Furthermore, the highest concentration of fatty acids in JSWR-1 FBC was hexadecanoic acid (16-carbon chain). Hexadecanoic acid (palmitic acid) is the predominant fatty acid in microorganisms. long-chain saturable palmitic acid (steric acid, C18:0) with a ratio of 11.37 percent. The fatty acid with the lowest concentration was erucic acid, with a ratio of 0.803%. The primary fatty acids C16 and C15 have been identified in the *Flavobacterium* genera [77]. However, unsaturated fatty acids distinguished the *Flavobacterium* genus from those previously reported [78-80]. C16:1 is the predominant fatty acid in 50.06 percent of JSWR-1, whereas C15 is the predominant fatty acid found in *Flavobacterium* species isolated from marine sediments [81]. Carotenoids accumulation in microbes is positively correlated with (C16, C18,3n3, C14:0, and C15:0) under various abiotic stress conditions. The number of saturated fatty acids increased when JSWR-1 was incubated at a lower temperature. The cell membrane of cold-adapted *Flavobacterium* species was predominantly composed of branched, unsaturated fatty acids. It had a direct effect on the membranés fluidity and frigid adaptation. Additionally, comparable adaptations were observed in both mesophilic and thermophilic *Flavobacterium* strains [82]. Less monounsaturated fatty acid (C22:1n9) was detected in JSWR-1. Padhan *et al*. (2021) identified erucic acid (C22:1n9) as the main component in the extracellular pigment synthesis of psychrotolerant *Paeninacillus* sp. BPW19 [83].

Zeaxanthin is widely recognized for its potent antioxidant properties and ability to neutralize free radicals through multiple mechanisms. A recent investigation indicated that the isomer meso-zeaxanthin exhibits substantial antioxidant properties [25]. Zeaxanthin, characterized by its yellow color and oxygenation, provides people with various advantages, such as protecting against retinal degeneration and reducing the risk of cancer development. The chemical can counteract free radicals, demonstrate antioxidant characteristics, and reduce inflammation. The test samples, FBC and BC, have shown strong capacities to scavenge free radicals and reduce ferrous ions, indicating their high antioxidant activity. The IC_50_ values for FBC and BC in DPPH radical scavenging activity were 0.93 and 1.75 mg/ml, respectively. Furthermore, the FRAP assay has been employed to ascertain the antioxidant capability of FBC and BC extracts. Recent studies have revealed the capacity of zeaxanthin obtained from microorganisms to effectively eliminate DPPH radicals [83, 84]. A marine *Flavobacterium* species has been found to synthesize zeaxanthin, a carotenoid molecule that exhibits promising antioxidant effects. The antioxidant activity of *Kocuria* sp. DPPH, a type of marine bacteria, was found to be 67.99% at a concentration of 1,000 μg/ml [85]. The antioxidant efficacy of natural carotenoids relies on the quantity of conjugated double bonds they possess and the substituents at the end of the molecule, which aid in neutralizing singlet oxygen and scavenging free radicals. The food industry is placing more and more emphasis on using natural colors. Bacterial pigments are attractive for industrial applications due to their impressive stability and excellent toxicological profiles. The scarcity of these products can be attributable to the growing demand for natural food colorings. Applying microbial fermentation to the industrial manufacturing of raw food colorants offers numerous notable benefits.

## Supplemental Materials

Supplementary data for this paper are available on-line only at http://jmb.or.kr.



## Figures and Tables

**Fig. 6:**
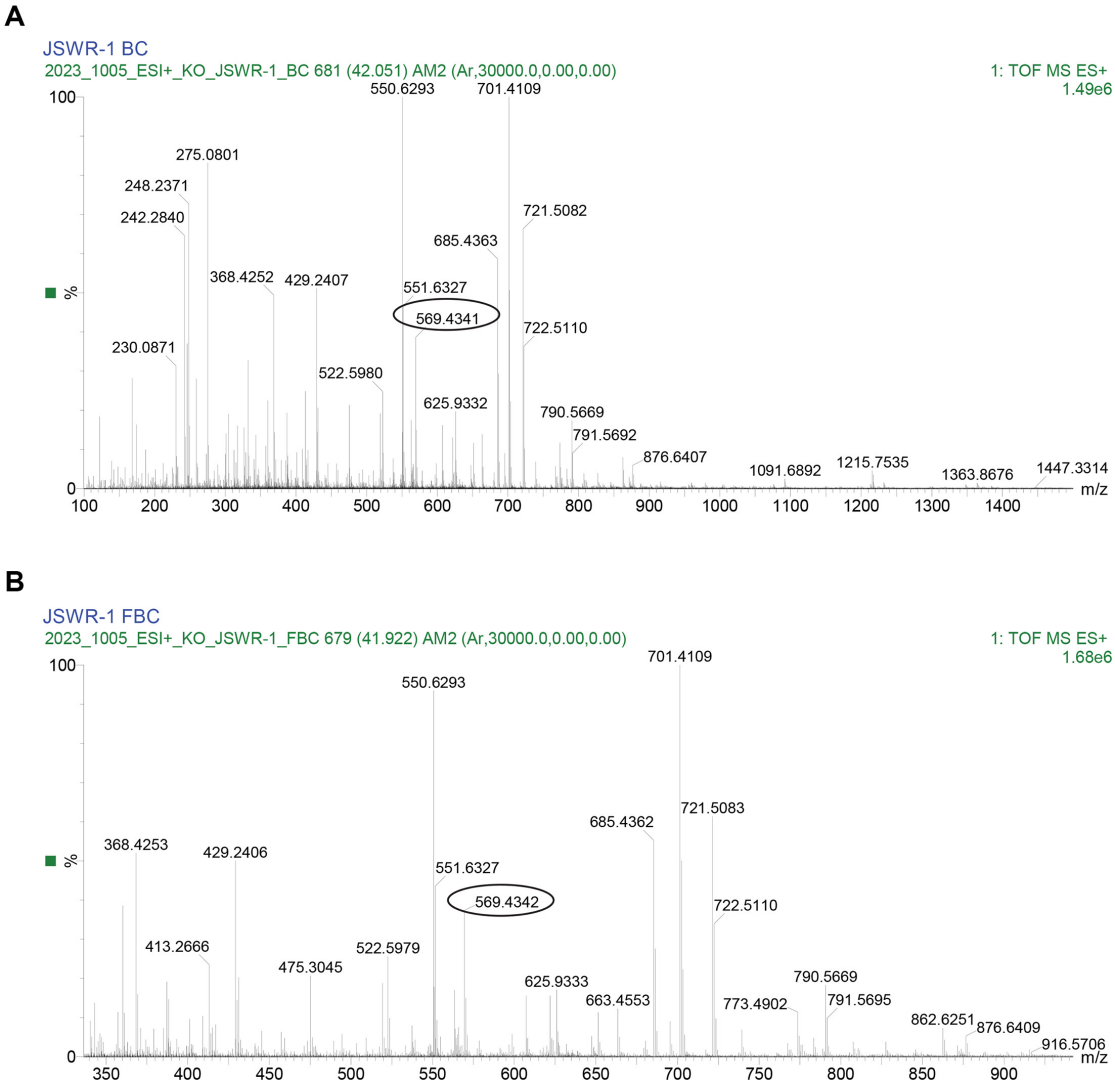
Results from identification of yellow carotenoids by liquid chromatography –mass spectrometer data. (**A**) BC, (**B**) FBC.

**Table 1:** Genomic features of *Flavobacterium* sp. JSWR-1.

Genome features	Chromosome
Genome size (bp)	3,425,829
Total number of Contigs	1
Genomic G + C content (%)	35.2
Coding sequences	2,941
Transfer-RNA genes	62
Ribosomal-RNA genes	15
Number of CDSs	2,941

**Table 2:** Effect of light sources on growth, total carotenoid and zeaxanthin in *Flavobacterium* sp. JSWR-1.

Incubation (h)	Growth OD_600_	Dry weight (mg/l)	Total carotenoid content (μg/g)	Zeaxanthin content (μg/g)	Zeaxanthin concentration (mg/l)
Red					
48	0.69 ± 0^d^	1.01 ± 0.08^bc^	397.03 ± 24.36^abc^	240 ± 0.01^cdefg^	3.51 ± 0.1^bc^
72	1.00 ± 0^f^	1.8 ± 0.23^efg^	469.06 ± 96.46^c^	264 ± 0^defghi^	5.34 ± 0.19^d^
96	1.36 ± 0^k^	2.79 ± 0.03^k^	330.78 ± 50.34^abc^	244 ± 0.20^cdefg^	7.14 ± 0.18^ef^
120	1.28 ± 0.01^j^	2.41 ± 0.01^hijk^	409.37 ± 3.61^abc^	326 ± 0.35^hijl^	7.84 ± 0.07^fg^
144	1.18 ± 0^h^	3.32 ± 0.05^l^	375.00 ± 14.79^abc^	239 ± 0^cdefg^	6.96 ± 0.15^ef^
Blue					
48	0.44 ± 0^a^	0.56 ± 0.02^ab^	469.69 ± 55.93^c^	319 ± 0.60^jijkl^	2.36 ± 0.22^a^
72	0.77 ± 01^e^	1.16 ± 0.09^cd^	416.88 ± 14.07^abc^	333 ± 0.13^ijkl^	4.26 ± 0.29^c^
96	0.99 ± 01^f^	1.48 ± 0.30^cde^	424.06 ± 8.30^bc^	255 ± 0.24^cdefgh^	6.12 ± 0.29^de^
120	1.18 ± 0^h^	2.13 ± 0.03^fghi^	386.25 ± 19.49^abc^	271 ± 0.28^efghij^	8.62 ± 0.32^ghi^
144	1.29 ± 0^j^	2.3 ± 0.09^ghijk^	328.75 ± 25.54^abc^	201 ± 0.40^abcde^	8.49 ± 1.06^gh^
Green					
48	0.38 ± 0^a^	0.5 ± 0.05^ab^	396.41 ± 40.96^abc^	145 ± 0.18^a^	1.90 ± 0.05^a^
72	0.75 ± 0^de^	1.63 ± 0.17^def^	343.12 ± 32.84^abc^	147 ± 0.28^a^	4.03 ± 0.12^c^
96	1.21 ± 01^hi^	2.17 ± 0.15^ghi^	300.00 ± 32.84^ab^	149 ± 0.01^a^	5.45 ± 0.51^d^
120	1.26 ± 0^ij^	2.2 ± 0.35^ghij^	298.59 ± 43.84^ab^	167 ± 0.07^ab^	6.39 ± 0.44^de^
144	1.20 ± 01^hi^	2.45 ± 0.17^ijk^	308.59 ± 9.92^ab^	154 ± 0.12^ab^	9.64 ± 0.45^ij^
White					
48	0.51 ± 0.03^b^	0.29 ± 0.03^a^	385.94 ± 92.02^abc^	198 ± 0.14^abcd^	2.66 ± 0.07^ab^
72	1.10 ± 0.01^g^	1.89 ± 0.22^efgh^	563.83 ± 32.75^c^	337 ± 0.23^jkl^	7.76 ± 0.27^fg^
96	1.26 ± 0.01^ij^	2.59 ± 0.13^ijk^	368.75 ± 54.49^abc^	302 ± 0.63^ghijk^	9.48 ± 0.03^hij^
120	1.20 ± 0^hi^	2.52 ± 0.09^ijk^	443.91 ± 27.97^bc^	443 ± 0.08^m^	12.80 ± 0.02^m^
144	1.16 ± 0^gh^	2.28 ± 0.02^ghijk^	270.31 ± 12.99^a^	224 ± 0.14^bcdef^	11.93 ± 0.58^lm^
Dark					
48	0.58 ± 01^c^	1 ± 0.30^bc^	330.31 ± 53.40^abc^	190 ± 0.14^abc^	2.42 ± 0.40^a^
72	1.16 ± 01^gh^	1.79 ± 0.10^efg^	384.69 ± 39.33^c^	285 ± 0.14^fghij^	6.58 ± 0.76^e^
96	1.31 ± 0^jk^	2.53 ± 0.01^ijk^	393.91 ± 2.35^abc^	279 ± 0.02^fgjij^	9.22 ± 0.36^hi^
120	1.29 ± 01^j^	2.74 ± 0.05^jk^	458.13 ± 42.94^c^	373 ± 0.24^kl^	11.14 ± 0.05^kl^
144	1.33 ± 01^jk^	2.53 ± 0.57^ijk^	391.41 ± 7.04^abc^	381 ± 0.13^lm^	10.35 ± 0.47^jk^

Light source continues exposure; Growth observed at 600 nm; initial pH 8.5; temperature 10°C; in rotary shaker (120 rpm); cultivation period days (48-144 h). The column and rows represent the mean value ± SD (*n* = 3) respectively, different alphabetic letters indicate significant difference *p* < 0.05 by Duncan's multiple range tests.

**Table 3:** Production of zeaxanthin by bacteria species primarily belonging to the genera *Flavobacterium* and *Paracoccus*.

Strains	Sources	Growth medium	Conditions [T, rpm]	Incubation time (h)	Zeaxanthin [mg/L]	References
*F. multivorum*	Water, High Ridge, Mo, USA	Complex media	37, -	48	190	71
*F. multivorum*	ATCC 55238	Basal liquid media	30, 250	44	9.8	60
*F. multivorum*	Mutant AFB-44 ATCC 55238	Complex media	30, 250	32	512	34
*Flavobacterium* sp.	ATCC 25582	Chemical defined medium	29, 120	48	1	62
*F. multivorum*	ATCC 55238	Complex medium with supplement	30, 250	60	10.65	25
*Flavobacterium* sp.	(ATCC 21588)	Bioreactor,4.6% corn steep liquor	-, 600	72	9.8	26
*Flavobacterium* sp. JSWR-1	Water, South Korea	Minimal media with supplement with glucose and yeast extract	10, 200	120	**16.69**	**Present study**
*Muricauda lutaonensis*	KCTC 22339T	Marine broth (Difco 2216).	-, 40	-	3.12	72
*Pseudomonas putida*	KT2440, recombinant	Luria–Bertani and terrific broth	25, 160	48-72	51	73
*Paracoccus zeaxanthinifaciens*	ATCC 21588, Ghent University, Belgium	The OVAT based production medium	30, -	72	11.63	27
*P. zeaxanthinifaciens*	ATCC 21588, Belgium.	Defined production media	30, 180	24	14.79	74
*Yarrowia lipolytica*	PO1f (ATCC MYA-2613)	Yeast extract peptone dextrose	28, 200	48	21.98	75
