# Supplementary material for: Overproduction of Xanthophyll Pigment in Flavobacterium sp. JSWR-1 under Optimized Culture Conditions
Source: J Microbiol Biotechnol. 2023 Nov 24;34(3):710–24. doi: 10.4014/jmb.2310.10034 (PMC11016774; doi:10.4014/jmb.2310.10034)

J. Microbiol. Biotechnol.
https://doi.org/10.4014/jmb.2310.10034

Supplementary Tables and Figures

Enhanced the production of yellow-orange carotenoid by *Flavobacterium* sp. JSWR-1 under optimized culture conditions

Jegadeesh Raman, Young-Joon Ko, Soo-Jin Kim*

Agricultural Microbiology Division, National Institute of Agricultural Sciences, Rural Development Administration, Wanju-gun, Jeollabuk-do 55365, Republic of Korea

Soo-Jin Kim, sinhye@korea.kr

Table S1. Variable carbon and nitrogen sources for *Flavobacterium* sp. JSWR-1 production of biomass and yellow-orange carotenoid. After sterilizing the media, an equal amount of $MgSo_4$ was added; All constituents exhibit a concentration expressed in units of grams per liter (g/L).

**Conditions**	**Glucose**	**Yeast extract**	**$MgSo_4$**
Con 1	5	0	0.7
Con 2	0	5	0.7
Con 3	5	5	0.7
Con 4	10	5	0.7
Con 5	10	10	0.7
Con 6	10	15	0.7
Con 7	15	5	0.7
Con 8	15	10	0.7
Con 9	15	15	0.7
Con 10	20	5	0.7
Con 11	20	10	0.7
Con 12	40	5	0.7
Con 13	40	10	0.7

Fig. S1. **Distribution of COG functional annotation of *Flavobacterium* sp. JSWR-1.**

Bars indicate number of genes assigned to COG categories.

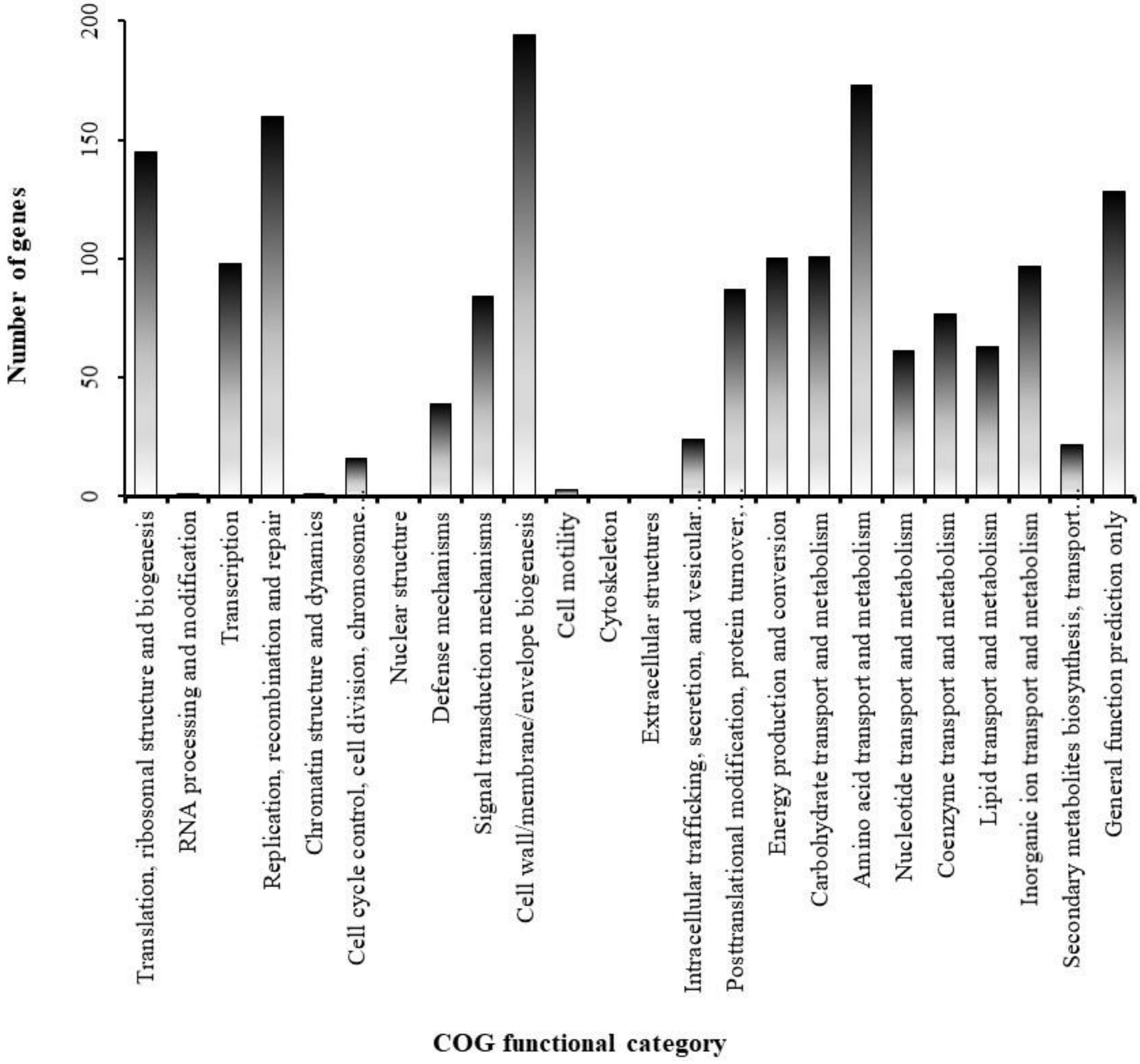

Fig. S2. **Phylogenetic tree constructed using the neighbor-joining method based on 16S rRNA gene sequences of closely related species within the same genus as *Flavobacterium* sp. JSWR-1.**

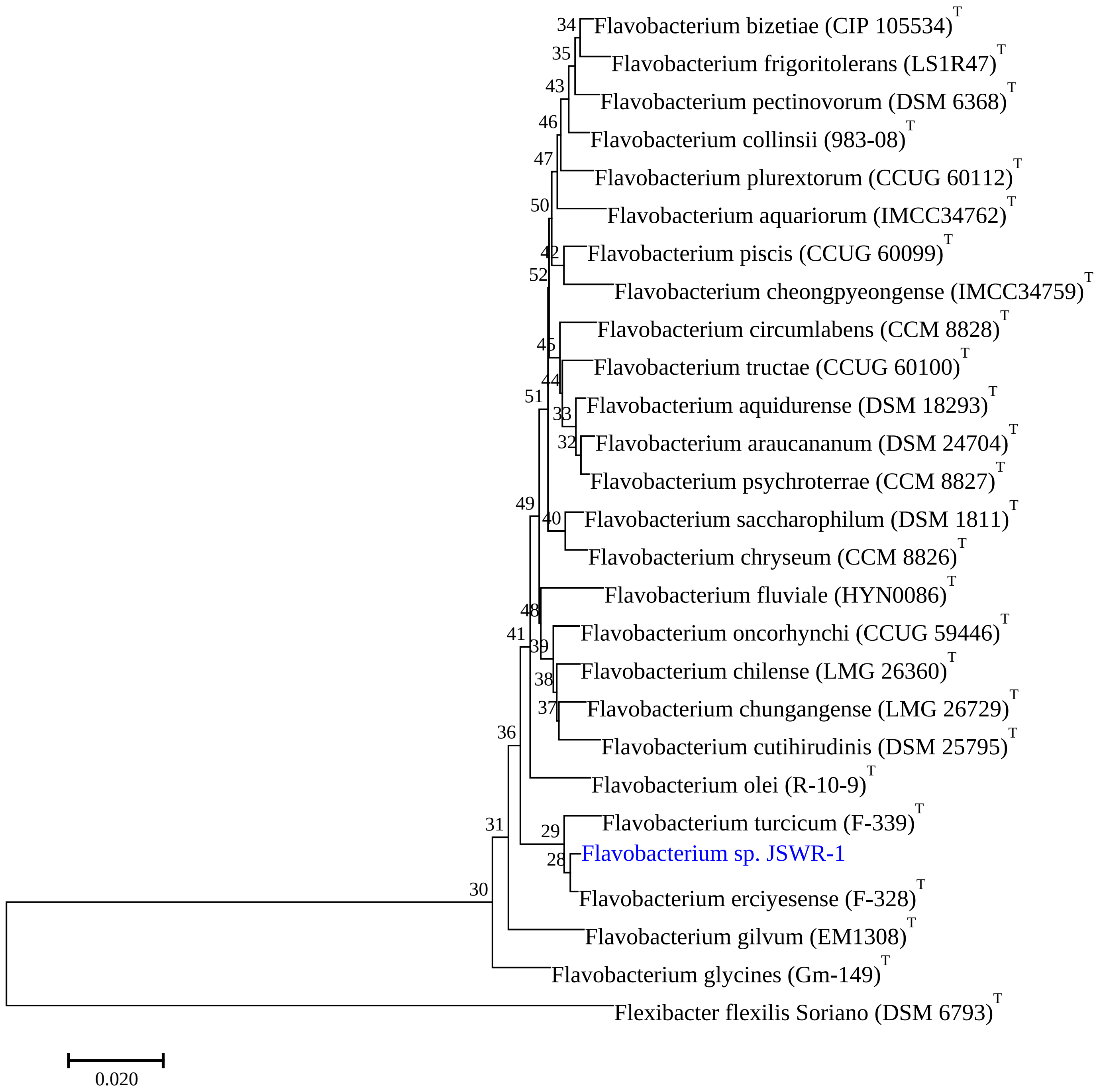

Fig. S3. Effect of temperature on growth and zeaxanthin production by *Flavobacterium* sp. JSWR-1. (A) Culture density (OD_{600}) on R2A minimal media. **(B)** Dry cell weight (mg/L).

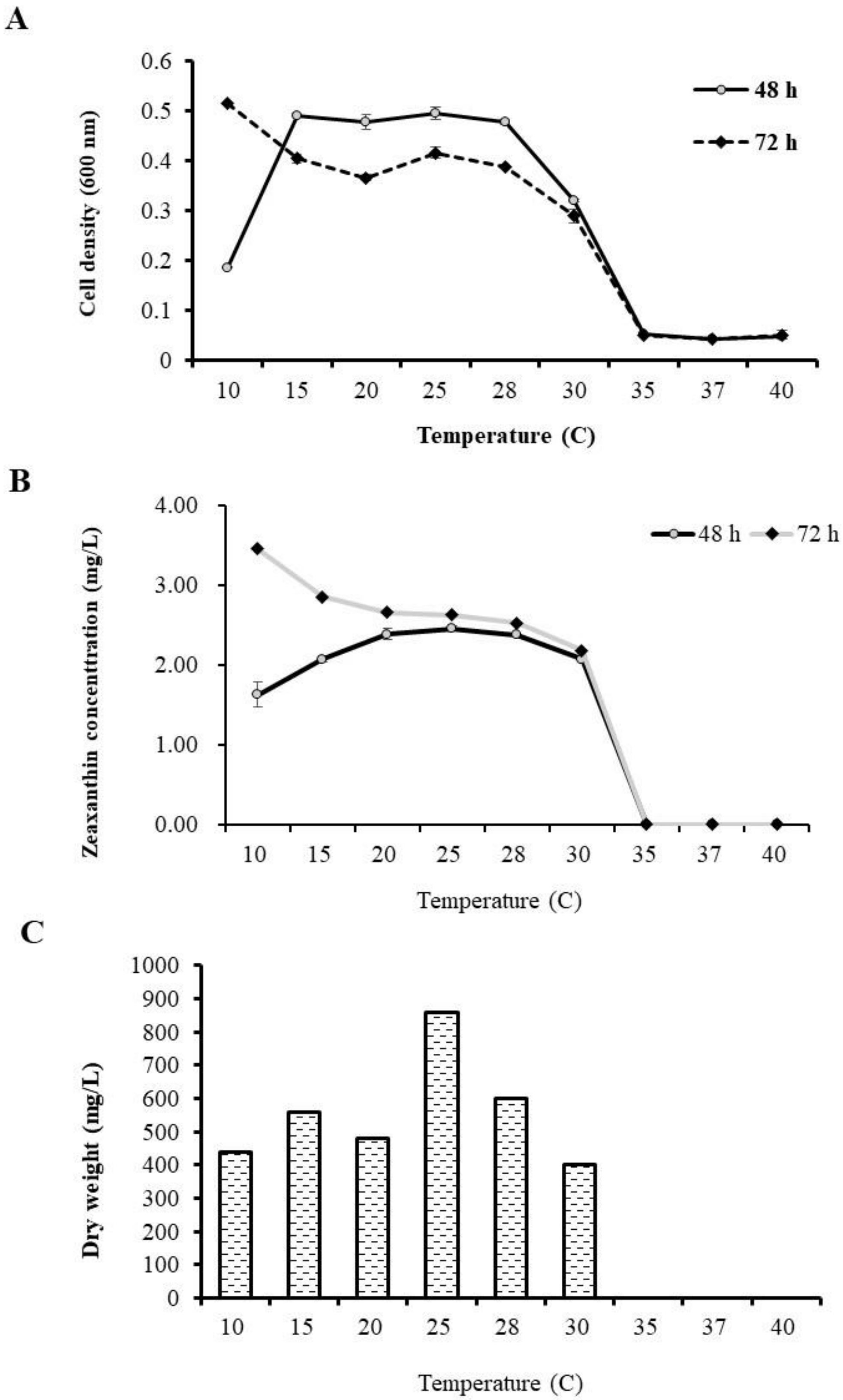
A
0.6
0.5
0.4
0.3
0.2
0.1
0
Cell density (600 nm)
48 h
72 h
10
15
20
25
28
30
35
37
40
Temperature (C)
B
4.00
3.00
2.00
1.00
0.00
Zeaxanthin concentration (mg/L)
48 h
72 h
10
15
20
25
28
30
35
37
40
Temperature (C)
C
1000
900
800
700
600
500
400
300
200
100
0
Dry weight (mg/L)
10
15
20
25
28
30
35
37
40
Temperature (C)

Fig. S4. Effect of pH on biomass production of JSWR-1 at 72 h incubation. Biomass was observed at 5 and 11 pH, pH 8 and 8.5 exhibit maximum dry biomass yield.

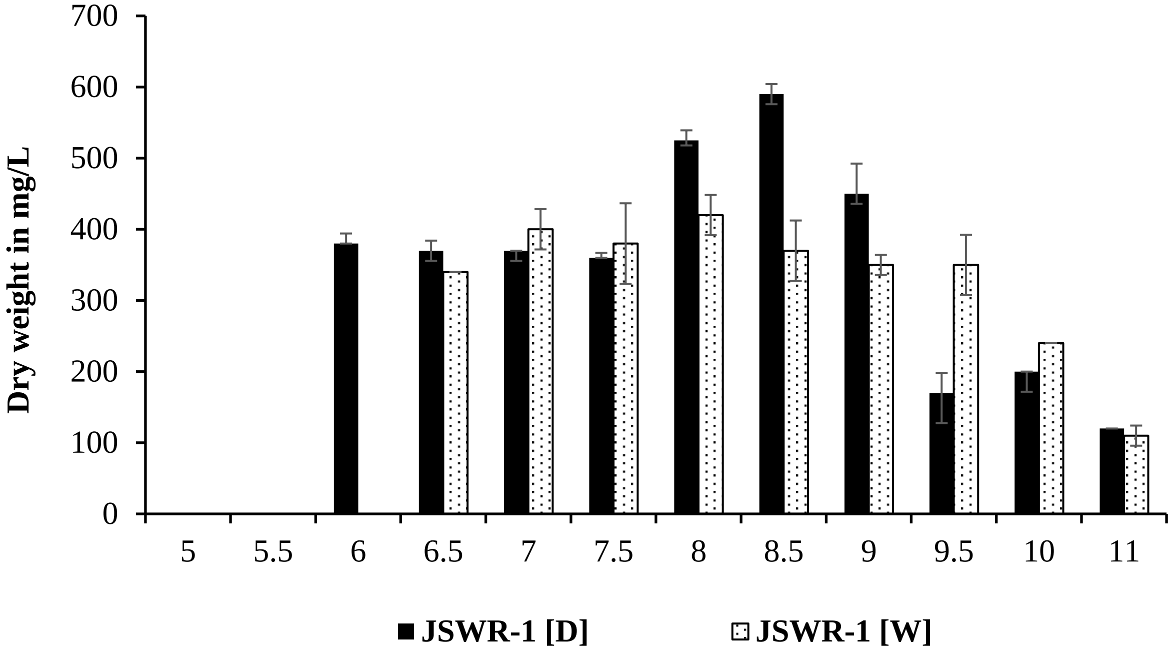

Fig. S5. LC-MS/MS profile of standard zeaxanthin.

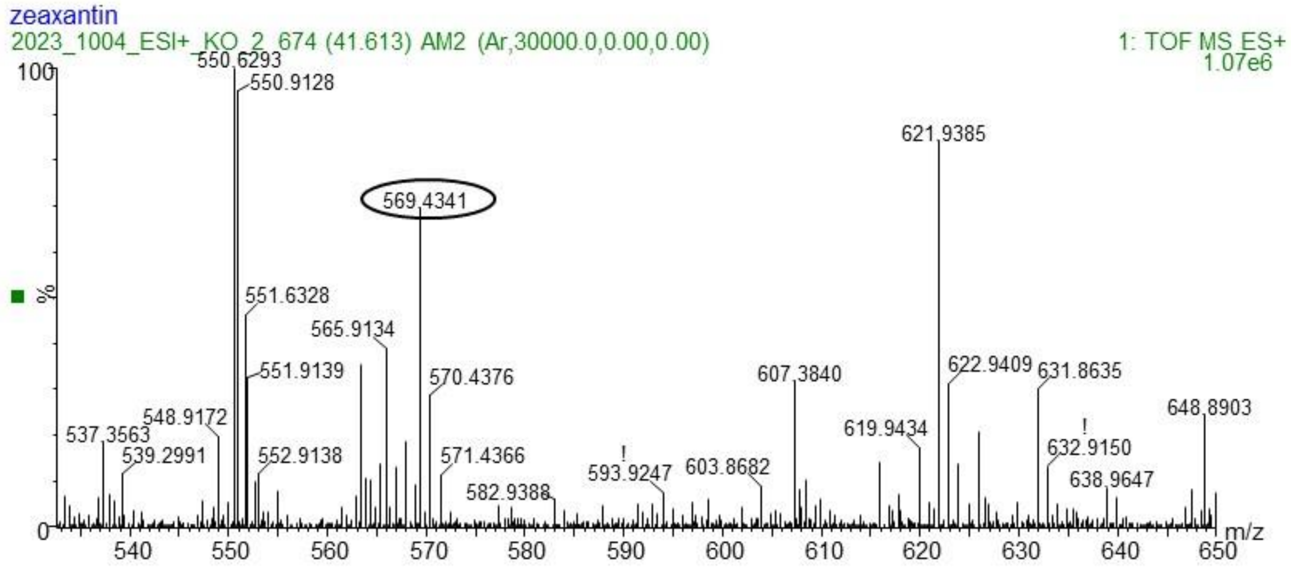

Fig. S6. Fatty acid profile of *Flavobacterium* sp. JSWR-1.

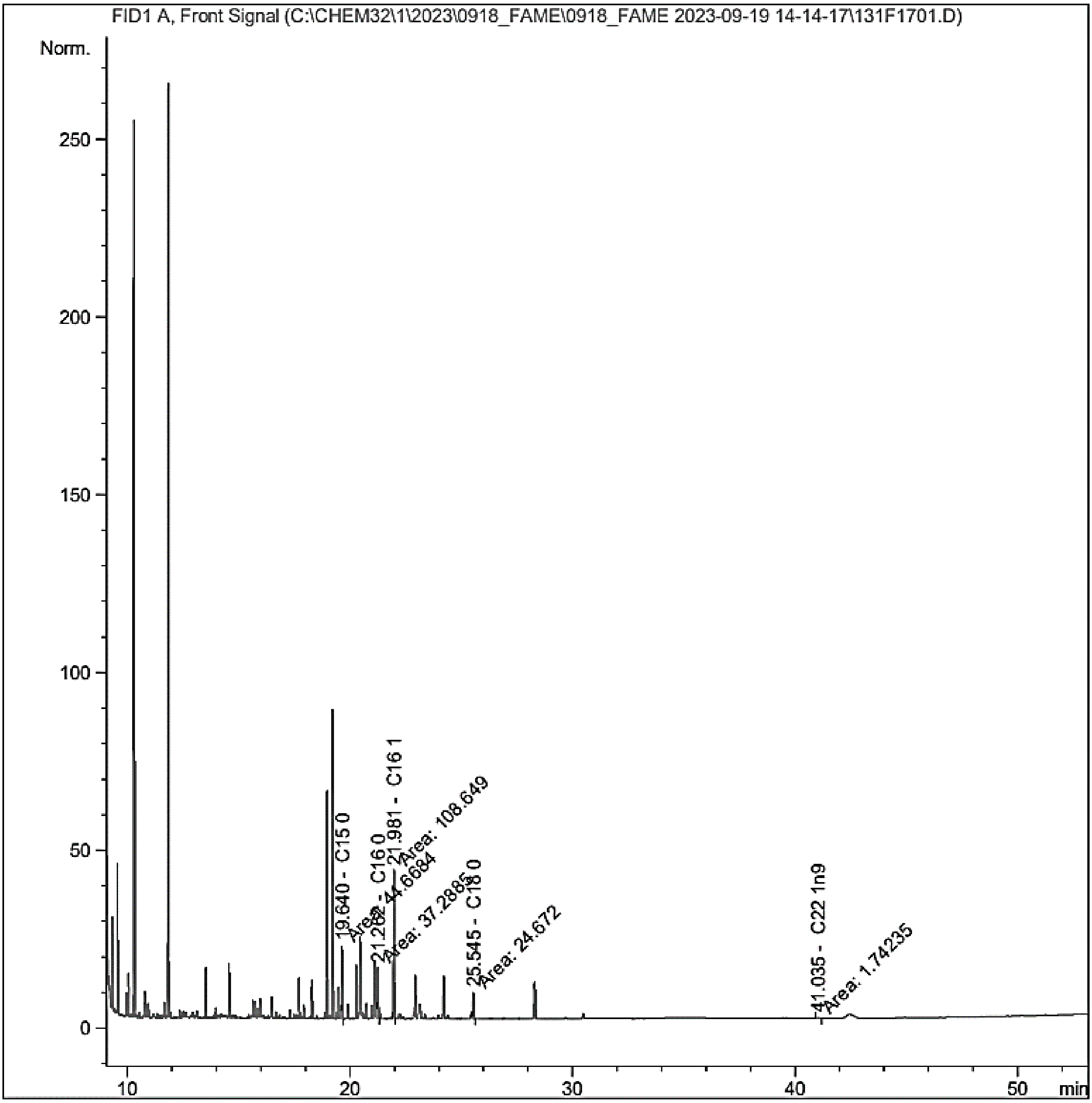

Fig. S7. DPPH radical scavenging assay. IC_{50} value of zeaxanthin and ascorbic acid. **(A)** Zeaxanthin 82.74 µg mL^{-1}; **(B)** Ascorbic acid 12.30 µg mL^{-1}.

A

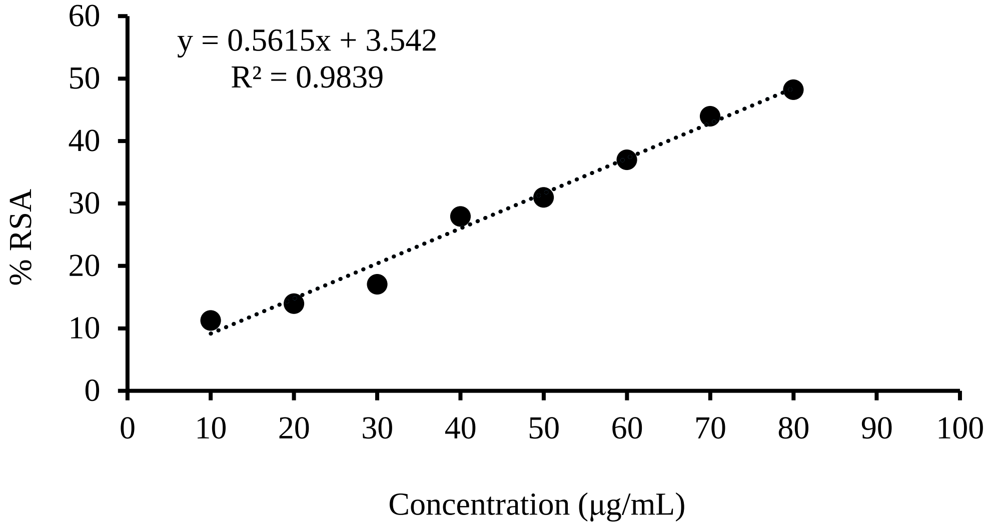

B

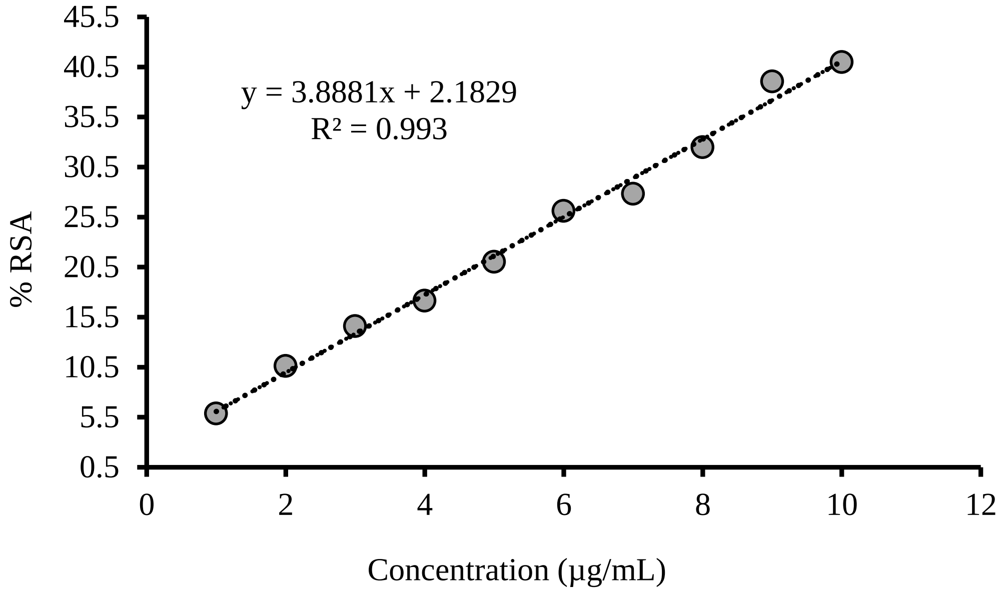

Supplement: Supplementary file 1 [file jmb-34-3-710-supple.pdf]
